# Terpene Glycosides from the Roots of *Sanguisorba officinalis* L. and Their Hemostatic Activities

**DOI:** 10.3390/molecules17077629

**Published:** 2012-06-25

**Authors:** Wei Sun, Zi-Long Zhang, Xin Liu, Shuang Zhang, Lu He, Zhe Wang, Guang-Shu Wang

**Affiliations:** School of Pharmaceutical Sciences, Jilin University, Changchun 130021, China

**Keywords:** *Sanguisorba officinalis*, ziyu-glycoside I, citronellol, geraniol, hemostasis

## Abstract

Guided by a hemostasis bioassay, seven terpene glycosides were isolated from the roots of *Sanguisorba officinalis* L. by silica gel column chromatography and preparative HPLC. On the grounds of chemical and spectroscopic methods, their structures were identified as citronellol-1-*O*-α-L-arabinofuranosyl-(1→6)-β-D-glucopyranoside (**1**), geraniol-1-*O*-α-L-arabinofuranosyl-(1→6)-β-D-glucopyranoside (**2**), geraniol-1-*O*-α-L-arabinopyranosyl-(1→6)-β-D-glucopyranoside (**3**), 3β-[(α-L-arabinopyranosyl)oxy]-19α-hydroxyolean-12-en-28-oic acid 28-β-D-glucopyranoside (**4**), 3β-[(α-L-arabinopyranosyl)-oxy]-19α-hydroxyurs-12-en-28-oic acid 28-β-D-glucopyranoside (ziyu-glycoside I, **5**), 3β,19α-hydroxyolean-12-en-28-oic acid 28-β-D-glucopyranoside (**6**) and 3β,19α-di-hydroxyurs-12-en-28-oic acid 28-β-D-glucopyranoside (**7**). Compound **1** is a new mono-terpene glycoside and compounds **2**, **3 **and **5** were isolated from the *Sanguisorba* genus for the first time. Compounds **1**–**7** were assayed for their hemostatic activities with a Goat Anti-Human α2-plasmin inhibitor ELISA kit, and ziyu-glycoside I (**5**) showed the strongest hemostatic activity among the seven terpene glycosides. This is the first report that ziyu-glycoside I has strong hemostatic activity.

## 1. Introduction

*Sanguisorba officinalis* L. (Rosaceae) is a perennial plant widely distributed in China, and its roots have been used as a traditional Chinese medicine for the treatment of hemostasis and inflammation [1]. A variety of chemical constituents, including tannins, triterpenoids, flavonoids, anthraquinones, steroids were isolated from *S. officinalis* L., and pharmacological studies on its hemostatic and anti-inflammatory properties have been reported [2,3], but the molecular level mechanisms of these activities have not been reported until now. In order to study the mechanism of hemostasis, we have carried out the bioassay-guided isolation and identification of hemostatic constituents of the roots of *S. officinalis* L. In the present study, we report the isolation and identification of a new monoterpene glycoside **1**, together with two known monoterpene glycosides **2**, **3** and four known triterpenoids **4**–**7**, and their hemostatic activities.

## 2. Results and Discussion

**Figure 1:**
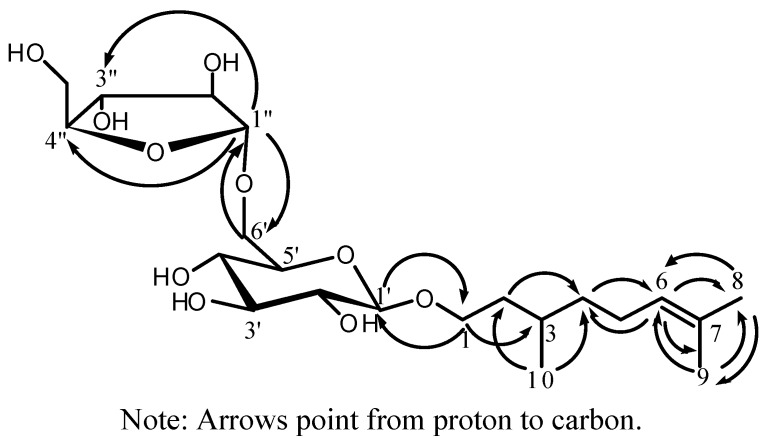
The key HMBC correlations of compound **1**.

Compound **1**, a colorless amorphous powder, produced a positive reaction to Molish reagent, and had the molecular formula C_21_H_38_O_10_ as determined by HRESIMS ([M+Na]^+^ *m/z *473.2369). Its IR spectrum indicated the presence of hydroxyl (3450 cm^−1^) groups. Acid hydrolysis of compound **1** gave D-glucose and L-arabinose. The ^1^H-, ^13^C- and DEPT-NMR spectrum (DMSO-d_6_) of compound **1** showed signals of a monoterpene moiety consisting of two singlets and one double methyl groups [δ_H_ 1.64 (3H, s, H-8), 1.56 (3H, s, H-9), and 0.85 (3H, d, *J* = 6.4 Hz H-10); δ_C_ 25.5 (C-8), 17.5 (C-9), and 19.3 (C-10)], four methylenes [δ_H_ 3.41 and 3.76 (each 1H, m, H-1), 1.32 and 1.56 (each 1H, m, H-2), 1.12 and 1.29 (each 1H, m, H-4), and 1.93 (2H, m, H-5); δ_C_ 66.9 (C-1), 36.3 (C-2), 36.8 (C-4), and 24.9 (C-5)], one olefinic methine [δ_H_ 1.52 (1H, m, H-3); δ_C_ 28.9 (C-3)], and one carbon-carbon double bond [δ_H_ 5.09 (1H, t-like, *J* = 7.2 Hz, H-6); δ_C_ 124.7 (C-6) and 130.4 (C-4)]. Based on the 2D NMR data of H-H COSY, HMQC and HMBC experiments, the monoterpene moiety was identified as citronellol [4]. The coupling constant of the anomeric proton of glucose at δ 4.11 (d, 1H, *J* = 8.0 Hz) indicated that glucose moiety was in a β-configuration. The HMBC correlation signal of the anomeric proton of glucose, 1'-H (δ_H_ 4.11) to C-1 (δ_C_ 66.9) showed that glucose was linked to C-1 of the alycone. The downfield shift by about 5 ppm of the signal of C-6' of glucose (δ_C_ 67.2) showed that arabinose was linked to C-6' of the glucose, which was further confirmed by the HMBC correlations of 6'-H (δ_H_ 3.39 and 3.85) to C-1" (δ_C_ 108.5) of arabinose and 1"-H (δ_H_ 4.79) to C-6' (δ_C_ 67.2). The ^13^C-NMR signals of compound **1** assignable to the arabinose moiety [δ_C_ 82.0 (C-2"), 83.8 (C-2")] and the coupling constant of the anomeric proton of arabinose at δ_H_ 4.79 (d, 1H, *J* = 1.6 Hz) indicated that arabinose moiety was an α- L -arabinofuranose moiety [5], which was further confirmed by the HMBC correlation of 1"-H (δ_H_ 4.79) to C-4" (δ_C_ 83.8). The complete assignment of the signals of compound **1** was based on DEPT ^13^C-NMR and 2D-NMR H-H COSY, HMQC and HMBC data. For all the ^1^H-, ^13^C-, and HMBC NMR data of compound **1** see Table 1, and for the structure of compound **1**, see Figure 1. Therefore, the structure of compound **1** was elucidated as citronellol-1-O-α-L-arabinofuranosyl-(1→6)-α-D-glucopyranoside.

Using similar methods as described above, compounds **2**–**7** were identified as geraniol-1-*O*-α-L-arabinofuranosyl-(1→6)-β-D-glucopyranoside (**2**) [5], geraniol-1-*O*-α-L-arabinopyranosyl-(1→6)-β-D-glucopyranoside (**3**) [5], 3β-[(α-L-arabinopyranosyl)oxy]-19α-hydroxyolean-12-en-28-oic acid 28-β-D-glucopyranoside (**4**) [6], 3β-[(α-L-arabinopyranosyl)oxy]-19α-hydroxyurs-12-en-28-oic acid 28-β-D-glucopyranoside (ziyu-glycoside І, **5**) [7], 3β,19α-hydroxyolean-12-en-28-oic acid 28-β-D-gluco-pyranoside (**6**) [8], 3β,19α-dihydroxyurs-12-en-28-oic acid 28-β-D-glucopyranoside (**7**) [8], respectively.

**Table 1:** ^1^H-NMR (400 MHz), ^13^C-NMR (100 MHz), HMQC and HMBC data of compound **1** (DMSO-d_6_, δppm).

No.	δC	δH	HMBC(H→C)	No.	δC	δH	HMBC(H→C)
aglycone				glc			
1	66.9	3.41, 3.76 (m, each 1H)	28.9, 102.8	1'	102.8	4.11 (d, 1H, *J* = 8.0 Hz)	66.9, 75.4, 76.7
2	36.3	1.32, 1.56 (m, each 1H)	19.3, 36.8,	2'	73.4	2.93 (t-like, 1H, *J* = 8.0 Hz)	
3	28.9	1.52 (m, 1H)	66.9	3'	76.7	3.13 (t, 1H, *J* = 8.8 Hz)	
4	36.8	1.12, 1.29 (m, each 1H)	19.3, 36.3, 124.7	4'	70.4	2.98 (t-like, 1H, *J* = 8.4 Hz)	73.4
5	24.9	1.93 (m, 2H)	28.9, 130.4	5'	75.4	3.28 (t-like, 1H, *J* = 8.4 Hz)	102.8
6	124.7	5.09 (t-like, 1H, *J* = 7.2 Hz)	17.5, 25.5, 36.8	6'	67.2	3.85 (d-like, 1H, *J* = 10.8 Hz), 3.39 (dd, 1H, *J *= 10.8, 8.4 Hz)	108.5
7	130.4			ara(f)			
8	25.5	1.64 (s, 3H)	17.5, 124.7	1"	108.5	4.79 (d, 1H, *J* = 1.6 Hz)	67.2, 77.2, 83.8
9	17.5	1.56 (s, 3H)	25.5, 124.7	2"	82.0	3.79 (m, 1H)	
10	19.3	0.85 (d, 3H, *J* = 6.4 Hz)	36.3, 36.8	3"	77.2	3.62 (m, 1H)	
				4"	83.8	3.72 (m, 1H)	
				5"	61.4	3.55 (dd, 1H, *J* = 11.6, 2.0 Hz), 3.40 (m, 1H)	

All assignments based on extensive 1D and 2D NMR experiments (HMQC, HMBC, 1H-1H COSY).

Compounds **1**–**7** were next assayed for hemostatic activity with a Goat Anti-Human α2-plasmin inhibitor ELISA kit, and the results are shown in Table 2. The data proved that ziyu-glycoside I showed strongest hemostasis activity within 7 kinds of terpene glycosides. It is the first report that ziyu-glycoside І (**5**) has strong hemostatic activity.

**Table 2:** The hemostasis assay data of the separated fractions and the isolated compounds.

**Fractions**	**H_2_O**	**30% EtOH**	**70% EtOH**	**95% EtOH**	**I**	**II**	**III**	**IV**	**Control blank**	**Standard**(10 g/L)
**OD value **(n = 3)	0.056 ± 0.011	0.053 ± 0.002	0.131 ± 0.014	0.051 ± 0.006	0.051 ± 0.005	0.051 ± 0.006	0.490 ± 0.017	0.061 ± 0.004	0.028 ± 0.004	0.828 ± 0.031
**Percent inhibition **	3.5	3.1	12.8	2.9	2.9	2.9	57.8	4.1		
**Compounds**	**1**	**2**	**3**	**4**	**5**	**6**	**7**		**Control blank**	**Standard**(10 g/L)
**OD value** (n = 3)	0.138 ± 0.016	0.111 ± 0.009	0.122 ± 0.003	0.260 ± 0.013	0.741 ± 0.012	0.227 ± 0.010	0.214 ± 0.015		0.060 ± 0.004	0.828 ± 0.031
**Percent inhibition**	10.2	6.6	8.1	26.0	88.7	21.7	20.1			

Note: Percent inhibition = [(OD_sample_ − OD_blank_) ÷ (OD_standard_ − OD_blank_)] × 100; the OD value is directly proportional the concentration of α2-plasmin inhibitor present in the samples.

## 3. Experimental

### 3.1. General

IR spectra were recorded on a FT-IR 5DX Nicolet/Nicolet Magna IR-560 spectrometer (Thermo Scientific, Osaka, Japan). ^1^H- and ^13^C-NMR spectra were recorded on a Bruker AV-400 spectrometer (Zürich, Switzerland). HR-ESI-MS were recorded on a Bruker microOTOF-Q II mass spectrometer. Prep. HPLC was performed on a Shimadzu LC-10A equipped with a SPD-10A detector and Gemini 5μm C18 110A column (250 mm × 10.00 mm, 5 μm, flow rate: 3.0 mL/min). The bioactivities were measured on a DG5033A Enzyme immunoassay spectrophotometer (Nanjing, China), using Goat Anti-Human α2-plasmin inhibitor ELISA kit [96/48-wells microtiter plates, plastic cover, standard (40 g/L), blank control, standard diluent, biotinylated anti-α2-plasmin inhibitor, streptavidin-HRP, washing buffer, substrate A, substrate B, stopping solution, sample diluent)] (R&D Systems, Minneapolis, MN, USA). The roots of *S. officinalis* L. were collected in Tong-Hua County in Jilin Province, China. They were identified by Prof. Jing-min Zhang of School of Pharmaceutical Sciences, Jilin University.

### 3.2. Extraction and Isolation

The air-dried the roots of *S. officinalis* (4.0 kg) were extracted with hot 70% EtOH (ca. 20 L, 24 h, 45 °C). The EtOH extract was concentrated under reduced pressure, and the viscous concentrate (420 g) was passed through a D101 polyporous resin column eluting successively with H_2_O, 30% EtOH, 70% EtOH, and 95% EtOH, and by vacuum distillation recovery, four fractions (I–IV) were obtained. The bioassay experiments suggested that the 70% EtOH portion had hemostatic activity. Therefore the 70% EtOH fraction was subjected to silica gel column chromatography eluted with a stepwise gradient mixture of CHCl_3_–MeOH (9:1; 6:1; 3:1), and finally with MeOH alone, and four fractionons (I–IV) were obtained. Fraction III having hemostasis activity was further subjected to a silica gel column eluted with CHCl_3_–MeOH–EtOAc–H_2_O (6.5:5:4:1.7), and three fractions (A, B, C) were obtained. Fraction B was applied to a ODS-A (50 μm, 12 nm, YMC, Kyoto, Japan) column eluted with a stepwise gradient mixture of MeOH-H_2_O (2:3; 3:2; 4:1), and thus compound **5** (230 mg) and Fraction D were obtained. Fractions A, C, and D were separated by preparative HPLC using MeOH–H_2_O (60:40, 70:30, 65:35, respectively), and compound **1** (22 mg), **2** (25 mg), and **3** (27 mg) were isolated from fraction A, compound **6** (30 mg) and **7** (33 mg) from fraction C, and compound **4** (30 mg) from Fraction B.

Compound **1**: Colorless amorphous powder, [α]^24^_D_: −21.0° (c 0.6, MeOH). HRESIMS, *m/z*: 473.2369 ([M+Na]^+^; calcd for C_21_H_38_O_10_Na, 473.2363). IR (KBr) ν_max_: 3450 cm^−1^. ^1^H and ^13^C-NMR: See Table 1.

Compound **2**: Colorless amorphous powder. HRESIMS, *m/z*: 471.2209 ([M+Na]^+^; calcd for C_21_H_36_O_10_Na, 471.2206). IR (KBr) ν_max_: 3440 cm^−^^1^. ^1^H-NMR (DMSO-d_6_) δ: 1.57 (s, 3H, H-9), 1.61 (s, 3H, H-10), 1.64 (s, 3H, H-8), 1.99 (2H, t, *J* = 6.8 Hz, H-4), 2.05 (2H, m, H-5), 4.08 (1H, dd, *J* = 11.9, 7.6 Hz, H-1a), 4.19(1H, dd, *J* = 11.9, 6.1 Hz, H-1b), 5.07 (1H, t-like, *J* = 6.8 Hz, H-6), 5.26 (1H, m, H-2); 4.11 (1H, d, *J* = 7.8 Hz, glc-H1), 2.95 (1H, t-like, *J* = 8.0 Hz, glc-H2), 3.12 (1H, t-like, *J* = 8.8 Hz, glc-H3), 2.98 (1H, t-like, *J* = 8.8 Hz, glc-H4), 3.25 (1H, t-like, *J* = 8.4 Hz, glc-H5), 3.86 (1H, d-like, *J* = 9.9 Hz, glc-H6a), 3.39 (1H, m, glc-H6b); 4.79 (1H, d, *J* = 1.6 Hz, ara-H1), 3.79 (1H, m, ara-H2), 3.62 (1H, m, ara-H3), 3.72 (1H, m, ara-H4), 3.52 (1H, d-like, *J* = 13.4Hz, ara-H5a),3.40 (m, 1H, ara-H5b). ^13^C-NMR (DMSO-d_6_) δ: 16.1(C-10), 17.6 (C-9), 25.6 (C-8), 25.9 (C-5), 39.4(C-4), 64.2 (C-1), 120.6 (C-2), 123.9 (C-6), 130.4 (C-7), 139.4 (C-3); 101.3 (glc-C1), 73.3 (glc-C2), 76.7 (glc-C3), 70.5 (glc-C4), 75.5 (glc-C5), 67.2 (glc-C6); 108.5 (ara(f)-C1), 82.1 (ara(f)-C2), 77.3 (ara(f)-C3), 83.8 (ara(f)-C4), 61.4 (ara(f)-C4).

Compound **3**: Colorless amorphous powder. HRESIMS, *m/z*: 471.2208 ([M+Na]^+^; calcd for C_21_H_36_O_10_Na, 471.2206). IR (KBr) ν_max_: 3445 cm^−1^. ^1^H-NMR (DMSO-d_6_) δ: 1.57 (s, 3H, H-9), 1.61 (s, 3H, H-10), 1.64 (s, 3H, H-8), 1.99 (2H, t, *J* = 6.8 Hz, H-4), 2.05 (2H, t, *J* = 6.8 Hz, H-5), 4.06 (1H, dd, *J* = 12.7, 7.3 Hz, H-1a), 4.22(1H, dd, *J* = 12.7, 6.0 Hz, H-1b), 5.08(1H, t-like, *J* = 6.5 Hz, H-6), 5.28 (1H, *J* = 6.5 Hz, H-2); 4.12 (1H, d, *J* = 7.8 Hz, glc-H1), 4.20 (1H, d, *J* = 6.0 Hz, ara-H1). ^13^C-NMR (DMSO-d_6_) δ: 16.1 (C-10), 17.6 (C-9), 25.5 (C-8), 25.8 (C-5), 39.3 (C-4), 64.4 (C-1), 120.7 (C-2), 123.9 (C-6), 130.9 (C-7), 139.1 (C-3); 101.5 (glc-C1), 73.3 (glc-C2), 76.6 (glc-C3), 70.5 (glc-C4), 75.6 (glc-C5), 67.2 (glc-C6); 103.4 (ara(p)-C1), 70.1 (ara(p)-C2), 72.5 (ara(p)-C3), 68.5(ara(p)-C4), 64.7 (ara(p)-C4).

Compound **4**: Colorless amorphous powder. HRESIMS, *m/z*: 789.4404 ([M+Na]^+^; calcd for C_41_H_66_O_13_Na, 789.4401). IR (KBr) ν_max_: 3440, 1720 cm^−1^. ^1^H-NMR (DMSO-d_6_) δ: 5.24 (1H, d, *J* = 7.8 Hz, glc-H1), 5.23(1H, br.s, H-12), 4.45 (1H, d, *J* = 7.0Hz, ara-H1), 3.11 (1H, br.s, H-19), 3.01 (1H, dd, *J* = 11.5, 3.9 Hz, H-3), 2.91(1H, br.s, H-18), 1.23 (3H, s, Me-27), 0.97 (3H, s, Me-23), 0.87 (3H × 2, s, Me-26 and Me-29), 0.85 (3H, s, Me-30), 0.76 (3H, s, Me-24), 0.65(3H, s, Me-25). ^13^C-NMR (DMSO-d_6_) δ: 38.0(C-1), 25.6 (C-2), 87.7 (C-3), 38.8 (C-4), 55.1 (C-5), 17.9 (C-6), 32.3 (C-7), 40.2 (C-8), 47.3 (C-9), 36.4 (C-10); 23.2 (C-11), 122.3 (C-12), 143.2 (C-13), 41.1 (C-14), 28.3 (C-15), 27.0 (C-16); 45.2 (C-17), 43.2 (C-18), 80.0 (C-19), 34.8 (C-20), 27.8 (C-21), 31.8 (C-22), 27.6 (C-23), 16.4 (C-24), 15.1 (C-25), 16.6 (C-26); 24.5 (C-27), 175.8 (C-28), 28.0 (C-29), 24.0 (C-30); 94.1 (glc-C1), 72.4 (glc-C2), 76.7 (glc-C3), 69.5 (glc-C4), 77.7 (glc-C5), 60.6 (glc-C6); 105.8 (ara(p)-C1), 71.0 (ara(p)-C2), 72.7 (ara(p)-C3), 67.6 (ara-C4), 65.1 (ara-C4).

Compound **5**: Colorless amorphous powder. HRESIMS, *m/z*: 789.4403 ([M+Na]^+^; calcd for C_41_H_66_O_13_Na, 789.4401). IR (KBr) ν_max_: 3475, 1740 cm^−1^. ^1^H-NMR (DMSO-d_6_) δ: 5.16 (1H, d, *J* = 7.8 Hz, glc-H1), 5.17(1H, br.s, H-12), 4.12 (1H, d, *J* = 6.0Hz, ara-H1), 3.11 (1H, br.s, H-19), 3.01 (1H, dd, *J* = 11.1, 3.9 Hz, H-3), 2.36(1H, br.s, H-18), 1.23 (3H, s, Me-27), 1.09 (3H, s, Me-29), 0.97 (3H, s, Me-23), 0.88 (3H, s, Me-26), 0.84(3H, d, *J* = 6.6 Hz, Me-30), 0.76 (3H, s, Me-25), 0.67 (3H, s, Me-24). ^13^C-NMR (DMSO-d_6_) δ: 38.2(C-1), 25.7 (C-2), 87.8 (C-3), 38.7 (C-4), 55.0 (C-5), 17.9 (C-6), 32.5 (C-7), 39.4 (C-8), 46.7 (C-9), 36.2 (C-10); 23.2 (C-11), 127.0 (C-12), 138.2 (C-13), 41.2 (C-14), 28.1 (C-15), 25.1 (C-16); 47.3 (C-17), 53.2 (C-18), 71.6 (C-19), 41.0 (C-20), 25.8 (C-21), 36.6 (C-22), 27.6 (C-23), 16.4 (C-24), 15.2 (C-25), 16.4 (C-26); 23.8 (C-27), 175.5 (C-28), 26.4 (C-29), 16.2 (C-30); 94.1 (glc-C1), 72.2 (glc-C2), 76.7 (glc-C3), 69.5 (glc-C4), 77.6 (glc-C5), 60.6 (glc-C6); 105.8 (ara(p)-C1), 71.0 (ara(p)-C2), 72.7 (ara(p)-C3), 67.6 (ara-C4), 65.1 (ara-C4).

Compound **6**: Colorless amorphous powder. HRESIMS, *m/z*: 657.39783 ([M+Na]^+^; calcd for C_36_H_58_O_9_Na, 657.39785). IR (KBr) ν_max_: 3440, 1720 cm^−1^. ^1^H-NMR (DMSO-d_6_) δ: 5.23 (1H, d, *J* = 7.6 Hz, glc-H1), 5.23(1H, br.s, H-12), 3.11 (1H, br.s, H-19), 3.00 (1H, dd, *J* = 11.2, 3.9 Hz, H-3), 2.91(1H, br.s, H-18), 1.23 (3H, s, Me-27), 0.89 (3H, s, Me-23), 0.87 (3H, s, Me-29), 0.84 (3H × 2, s, Me-25 and Me-30), 0.68 (3H, s, Me-24), 0.64(3H, s, Me-25). ^13^C-NMR (DMSO-d_6_) δ: 38.0 (C-1), 26.9 (C-2), 77.7 (C-3), 38.4 (C-4), 54.9 (C-5), 18.1 (C-6), 32.3 (C-7), 40.2 (C-8), 47.3 (C-9), 36.7 (C-10); 23.2 (C-11), 122.3 (C-12), 143.2 (C-13), 41.1 (C-14), 28.3 (C-15), 27.0 (C-16); 45.2 (C-17), 43.1 (C-18), 80.0 (C-19), 34.8 (C-20), 27.8 (C-21), 31.8 (C-22), 28.3 (C-23), 15.9 (C-24), 15.0 (C-25), 16.6 (C-26); 24.1 (C-27), 175.8 (C-28), 28.0 (C-29), 24.5 (C-30); 94.1 (glc-C1), 72.4 (glc-C2), 76.7 (glc-C3), 69.5 (glc-C4), 76.9 (glc-C5), 60.6 (glc-C6).

Compound **7**: Colorless amorphous powder. HRESIMS, *m/z*: 657.39784 ([M+Na]^+^; calcd for C_36_H_58_O_9_Na, 657.39785). IR (KBr) ν_max_: 3475, 1740 cm^−1^. ^1^H-NMR (DMSO-d_6_) δ: 5.16 (1H, d, *J* = 8.0 Hz, glc-H1), 5.17(1H, br.s, H-12), 4.12 (1H, d, *J* = 6.0Hz, ara-H1), 3.11 (1H, br.s, H-19), 3.00 (1H, dd, *J* = 11.0, 3.9 Hz, H-3), 2.37(1H, br.s, H-18), 1.27 (3H, s, Me-27), 1.08 (3H, s, Me-29), 0.89 (3H, s, Me-23), 0.85 (3H, s, Me-26), 0.84 (3H, d, *J* = 6.6 Hz, Me-30), (3H, s,), 0.67 (3H × 2, s, Me-25 and Me-24). ^13^C-NMR (DMSO-d_6_) δ: 38.2 (C-1), 27.0 (C-2), 77.6 (C-3), 38.4 (C-4), 54.8 (C-5), 18.1 (C-6), 32.6 (C-7), 39.2 (C-8), 46.7 (C-9), 36.5 (C-10); 23.2 (C-11), 127.0 (C-12), 138.1 (C-13), 41.2 (C-14), 28.1 (C-15), 25.1 (C-16); 47.3 (C-17), 53.2 (C-18), 71.6 (C-19), 41.1 (C-20), 25.4 (C-21), 36.6 (C-22), 28.2 (C-23), 16.4 (C-24), 15.1 (C-25), 16.2 (C-26); 23.8 (C-27), 175.5 (C-28), 26.4 (C-29), 16.0 (C-30); 94.0 (glc-C1), 72.2 (glc-C2), 76.7 (glc-C3), 69.5 (glc-C4), 76.9 (glc-C5), 60.6 (glc-C6). 

### 3.3. Acid Hydrolysis of ***1–7***

Solutions of **1**–**7** (each 1.0 mg) in 0.5 M H_2_SO_4_ (2.0 mL) were heated under reflux for 3 h. After cooling, each reaction mixture was diluted with H_2_O, neutralized with BaCO_3_, then filtered. The solution was partitioned with EtOAc to give two layers. The aqueous layer was evaporated and then subjected to TLC analysis with authentic sugar samples using *n*-BuOH–MeOH–CHCl_3_–HOAc (12.5:4.5:9:1.5:1, detection with aniline-phthalic acid). Compounds **1**–**5** afforded D-glucose (R_f_ = 0.30) and L-arabinose (R_f_ = 0.36), and **6**–**7** gave D-glucose (R_f_ = 0.30).

### 3.4. Bioactivity Assay

The hemostasis assay was carried out by using a Goat Anti-Human α2-plasmin inhibitor kit which is a solid phase sandwich enzyme-linked quantitative immunoabsorbent assay (ELISA) with a purified antibody specific for α2-plasmin inhibitors. The above separated fractions and compounds were dissolved in dimethylsulfoxide (DMSO) (0.188 mg/μL for the fractions; 0.094 mg/μL for compounds), and were diluted with sample diluent (1:1). After an aliquot (50 μL) of the above samples or standards was added to each microplate well, a portion of biotinylated anti-α2-plasmin inhibitor (50 μL) was immediately added to each well, and the microplate was incubated for 1 h at 37 °C. After a wash with washing buffer, streptavidin-HRP was added, and the microplate was incubated for 30 min at 37 °C. Again, after a wash with washing buffer, substrate A and substrate B (50 μL each) were added to each well, and the microplate was incubated for 10 min at 37 °C. The enzyme-substrate reaction was stopped by quickly pipetting stopping solution (50 μL) into each well. The optical density absorbance (OD value) of each well was measured at 450 nm wavelength on a DG5033A enzyme immunoassay spectrophotometer. All the separated fractions and isolated compounds were tested for their hemostasis activities, and the results are summarized in Table 2.

The correlation of calibration curve test data is shown in Table 3. By using OD value as Y-axis(Y) and standards cocentration as X-axis (X), the linear regression equation, y = 0.0687x + 0.0317, r = 0.9997, was obtained. The results showed that the linear relation between OD value and concentration of standards were good and linearity domain of the measure is 0~40g/L. Therefore the OD value is directly proportional the concentration of α2-plasmin inhibitor present in the samples.

**Table 3:** The correlation of calibration curve test data.

Standards Concentration (g/L)	40	20	10	5.0	2.5	1.25	Control blank
OD	2.9404	1.5171	0.828	0.4905	0.2955	0.0895	0.0214

Note: Y = 0.0687x + 0.0317, r = 0.9997, linearity domain: 0~40 g/L; the OD value is directly proportional the concentration of α2-plasmin inhibitor present in the samples.

## 4. Conclusions

Compound **1** is a new monoterpene glycoside and compounds **2**, **3** and **5** were isolated from the *Sanguisorba* genus for the first time. Compounds **1**–**7** were assayed for their hemostatic activities with a Goat Anti-Human α2-plasmin inhibitor ELISA kit, and the hemostatic constituent of *S. officinalis* wasidentified as ziyu-glycoside I (**5**). This is the first report that ziyu-glycoside I has strong hemostatic activity.
